# Trajectories of posttraumatic stress symptoms during and after Narrative Exposure Therapy (NET) in refugees

**DOI:** 10.1186/s12888-020-02720-y

**Published:** 2020-06-17

**Authors:** Elisa Kaltenbach, Katharin Hermenau, Maggie Schauer, Katalin Dohrmann, Thomas Elbert, Inga Schalinski

**Affiliations:** 1 Department of Psychology, University of Konstanz, Konstanz, Germany; 2 Centre for Research in Family Health, IWK Health Centre, Halifax, NS Canada; 3 Vivo International e.V., Konstanz, Germany; 4 Charité – Universitätsmedizin Berlin, Corporate Member of Freie Universität Berlin, Humboldt-Universität zu Berlin, and Berlin Institute of Health (BIH), Institute of Medical Psychology, Berlin, Germany

**Keywords:** Trauma therapy, Symptom trajectories, Refugee, Imaginal exposure, PTSD

## Abstract

**Background:**

Trauma-focused therapy approaches are recommended as treatment for posttraumatic stress disorder (PTSD). This includes the treatment of trauma-related suffering in refugee populations. However, there is a lack of knowledge about symptom trajectories in refugees living in volatile conditions. This has led to fear of “retraumatisation” and general skepticism in clinicians concerning the use of exposure therapy.

**Methods:**

To test the relevance of this concern, we investigated PTSD symptom trajectories and potentially influencing factors during the course of Narrative Exposure Therapy (NET) in a refugee sample living in Germany. Refugees filled out the PTSD Checklist prior to each treatment session and also during follow-up interviews. Therapists continuously documented positive and negative life events as well as the content of the treatment sessions. Additionally, structured clinical interviews were conducted pre-treatment and at follow-up time points.

**Results:**

On average, clients presented with substantial decreases in PTSD symptoms already during and after NET. However, symptom trajectories differed and ranged from fast responders to slow responders to no immediate response during treatment. Importantly, a persistent worsening of symptoms was not observed, also not after exposure to the most distressing events. In contrast, stressful life experiences seemed to aggravate PTSD symptoms.

**Conclusions:**

Consistent with earlier studies, NET leads to clinically and behaviorally relevant reductions in PTSD symptoms both throughout and following treatment in refugees living in volatile conditions. Concerns about imaginal exposure in refugees were not substantiated. While stressful life events contributed to transient symptom increases, they weren’t found to prevent the overall effectiveness of NET.

**Trial registration:**

NCT02852616.

## Background

Poor mental health remains one of the major concerns in refugee populations worldwide [1]. The experience of traumatic events in the home country and during the flight as well as postmigrational stressors lead to substantial prevalence rates of trauma-related suffering, including posttraumatic stress disorder (PTSD), affective, and anxiety disorders [2–4]. These high rates of mental health issues remain elevated even after years of living in a host country [5, 6]. Depending on the cumulative exposure to traumatic stressors experienced by refugees, prevalences of PTSD remain substantial with one in three survivors in need of treatment [7, 8]. This is especially important as reviews on the naturalistic course of PTSD show a chronic or recurrent course in the majority of clients [9]. Recent studies in refugee populations confirm this by showing that PTSD symptoms often persist across extended periods of time – sometimes for one’s whole life – when no treatment is offered [10, 11].

Several approaches for treating PTSD have been developed – thereof, trauma-focused psychotherapy (TFP) approaches show the best long-term benefit [12, 13]. Among others, all TFP approaches include the imaginal exposure of the traumatic experiences [14]. Research on PTSD treatments in refugees found two TFP approaches which have shown sound evidence and are recommended for treatment: Narrative Exposure Therapy (NET) and trauma-focused cognitive-behavior therapy (TF-CBT) [8, 15–17]. NET [18] was especially developed for individuals with multiple traumatic experiences [19–21]. The etiological model of NET is based on the concept that repeated traumatic experiences form a so-called fear network, i.e. an associated memory of sensory, cognitive, emotional, and interoceptive representations. This means, if one memory component is activated, other components have a higher likelihood to be recalled as well. For instance, seeing a man in uniform in the host country may activate the feeling of threat in a war survivor (even if this man is actually a protective resource in the current situation) which then leads to more violence-related memories. With increasing traumatic experiences, the number of associations within the fear network continues to grow and becomes detached from the autobiographical context (e.g. the time and place of the happening) [6, 18, 22]. Through an activation of this fear network and its connection with the autobiographical context, the present memory structure becomes segmented again in memories of the particular events. Then, specific cues may still activate the recall of a particular traumatic event but no more the non-locatable horror and fear [18]. NET shows sustained effects on PTSD symptoms as well as on comorbid disorders and functioning [23–25]. Additionally, a child-friendly version of NET (KIDNET) has been found to be effective in refugee children and adolescents [26, 27].

Despite the evidence in favor of TFP, it is not commonly used in clinical practice due to a variety of perceived barriers: lack of training in TFP, concerns of patients’ decompensation, drop-out, and symptom exacerbation [28–30]. Specifically for refugees, postmigrational stressors (i.a. the insecure living conditions) are often named as a concern of clinicians to use TFP [8]. However, up-to-date evidence on TFP for other target groups did not find differences in dropout and non-response rates compared to other active treatments [31, 32]. For refugee populations, NET has proven its effectiveness even in volatile and insecure settings [19, 33–35].

Most psychotherapy research to date has focused on assessing symptoms before and after treatment – however, emerging research began to assess symptoms during treatment as well. By examining the overall course of symptoms during TFP, most studies report linear or quadratic symptom changes [36–38]. More recent studies have tried to unravel trajectories during therapy by allocating the individuals to groups. Different patterns have been found where most studies group individuals with (a) high symptom decreases at the beginning of therapy, (b) gradual symptom decreases, and (c) little to no symptom changes during the time of therapy [39–41]. Symptom exacerbations due to exposure therapy seem to be present in a small minority of clients only [42, 43]. However, studies on in vivo exposure treatment for anxiety disorders show that exposure sessions may produce physiological reactions reflecting higher perceived stress for both the client and the therapist compared to non-exposure sessions [44]. Research on predictors influencing the symptom trajectories or outcomes (e.g. sex, age, education, initial mental health symptoms) shows inconsistent results [39, 45, 46].

To-date, the majority of PTSD treatment studies examining symptom trajectories were conducted with veterans using TF-CBT, prolonged exposure therapy, or cognitive processing therapy as TFP approaches [40, 41, 47]. To our knowledge, there are no current studies that have systematically examined symptom trajectories of refugees during treatment. Until now, only one case study depicted the course of symptoms in 6 refugees during a combination of NET and physiotherapy [48]. Otherwise, treatment studies with refugees have concentrated on a few assessment time points of symptoms only [49–51]. These studies have demonstrated the influence of postmigrational stressors and stressful life events on mental health symptoms during therapy [50, 51].

Given the lack of research on the trajectories of mental health symptoms in refugees and during NET as well as clinicians’ concerns in using TFP in refugee populations, the current study tackles the following issues: (1) We examine trajectories of self-reported PTSD symptoms and identify distinct symptom courses during NET as well as its implications on treatment outcome. (2) We explore the influence of imaginal exposure to the most distressing events as well as treatment-unrelated life stressors on PTSD symptoms during NET.

## Method

### Design and procedure

The study was conducted between 2015 and 2018 at the Center of Excellence for Psychotraumatology (CEP), a specialized research center for asylum seekers and refugees in Germany. Refugees were either referred to the CEP by social workers, physicians, volunteers, or lawyers, or they were recruited from asylum accommodations in southern Germany. All were initially invited to a structured clinical interview for the assessment of the trauma history and mental health status in the context of other studies [52–54]. In case of mental health problems, the participant and the psychologist decided after the interview how to proceed. Participants fulfilling the criteria of PTSD were offered NET at the CEP. Additionally, other treatment options such as a referral to psychiatric or other psychotherapeutic care were presented. A selection of refugees who fulfilled the criteria of PTSD were invited to participate in the present study. Those who did not need or did not wish treatment were included in a parallel study [11]. Refugees being referred to other care options were not followed up.

In the course of the study, the clients’ symptoms were assessed in the following ways:

1. Structured clinical interviews: In addition to the initial clinical assessment (T0), clinical interviews were conducted 3 (F3) and 6 months (F6) after treatment. All interviews included the assessment of PTSD and depression symptoms.
2. Self-report: Clients filled in a questionnaire about their PTSD symptoms and daily functioning before each treatment session (T_1_ – T_end of treatment_) as well as 3 and 6 months (F3 & F6) after treatment. The duration varied between clients, with an estimated mean of 10–15 min. The number of filled-in questionnaires varied depending on the number of treatment sessions.

**Fig. 1:**
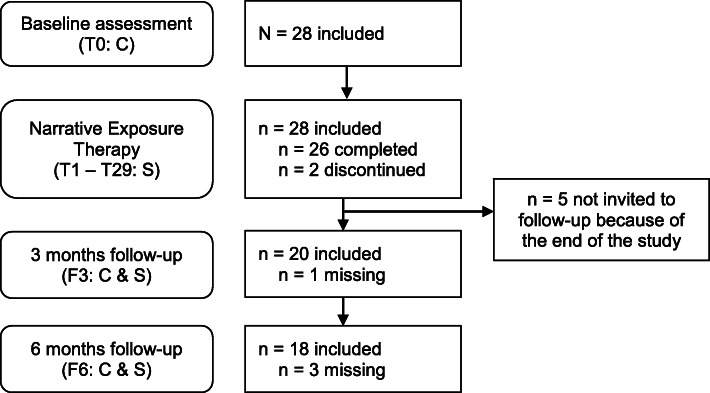
Flow of clients through the study. C = structured clinical interview, S = self-rating

Figure 1 shows the study design. The dropout rate during treatment was 7% (*n* = 2 of 28). One person dropped out because of lack of motivation for treatment (after session 4) and one without giving a reason (after session 1). Dropout for the follow-up interviews was 5% (*n* = 1 of 21) at F3 and 14% (*n* = 3 of 21) at F6. Five clients were not invited for the follow-up assessments because the follow-ups would have exceeded the end of the funding period. One client could not participate at F3 due to work reasons. At F6, reasons for dropout were relocation to the home country, the wish to end the participation, and lack of time (each *n* = 1).

The structured clinical interviews were conducted by 17 clinical psychologists with extensive experience in mental health diagnostics and working with refugee populations. Twelve clinical psychologists trained in NET and with substantial experience conducted the therapy. Training included a NET training workshop with theoretical and practical components, sitting in on other NET therapy sessions, and having an experienced NET therapist sitting in on the first cases. At all stages, supervision and intervision was provided. The majority of the interviews and therapy sessions (58%, *n* = 15) were conducted with the help of trained and experienced interpreters. The other interviews and therapy sessions were directly conducted by therapists either in German, English, or Farsi. Training of interpreters included an introduction session about how to translate in a mental health setting and self-care. Following the diagnostic and therapy sessions, interpreters were debriefed and received feedback. The clinical interviews at F3 and F6 were conducted by interviewers who did not know if the client received NET or was part of the concurrent non-treatment study [11].

Before the start of the interview and treatment, a comprehensive explanation of the objectives of the study and personal risks of participation were given. Furthermore, it was explained that the study is completely voluntary and no monetary compensation would be offered. All clients gave their written informed consent. In the case of minors, written informed consent was also given by the legal guardian. The study was approved by the Ethical Review Board of the University of Konstanz. The study was registered at Clinical Trials (clinicaltrials.gov) with the registration number NCT02852616 (08/01/2016).

### Treatment

The Narrative Exposure Therapy (NET) was conducted following the treatment manual of Schauer et al. (2011) [18]. At the beginning of the NET, the therapist gives psychoeducation on PTSD and an explanation of the rationale of NET. Then, the “lifeline” – a rope symbolizing the clients’ path of life – is used to gain an overview of the emotional experiences from birth to present and to structure the treatment. Thereby, highly arousing events with a positive valence are symbolized with flowers and the negative (fearful, traumatic, sad) ones with stones. In the following sessions, the client narrates his/ her life with the support of the therapist in a chronological order with focus on the most traumatic experiences. During narration, the therapist asks for sensory information, cognitions, emotions, physiological reactions, and meaning – both in the past and present when it is remembered – and connects them to the autobiographic context (time and place). After the sessions, the therapist writes down the narration and reads it to the client in the following session, before continuing with the narration of the next event. After completing the whole narration, the last session focuses on the client’s life with an outlook on the future. For this, the client again lays down the lifeline and the wishes for the future are added. At the end, the narration is handed out to the client.

Schauer et al. (2011) [18] recommend 8 to 12 sessions with 90 min each, noting that in very severe cases with complex trauma, even twice as many may be helpful. The aim of this study was to provide a naturalistic therapeutic setting, with the clinical impression of the need for more sessions overruling the recommended number of sessions.

### Sample

The sample consisted of 28 refugees living in Germany. Inclusion criteria were the status as a refugee or asylum seeker, age above 14 years, and fulfillment of the criteria of PTSD according to DSM-5. As we intended to conduct a naturalistic study, the only exclusion criteria were the presence of acute psychotic symptoms and acute suicidality leading to inpatient treatment. The two drop-outs are not included in the analyses. Clients were on average M = 28.8 years (SD = 12.2, range 14–61) old. Six of them were under the age of 18. Thirty-nine percent (*n* = 10 of 26) were female. The majority of refugees in this sample came from Afghanistan (42%; *n* = 11 of 26), Western Asian countries (27%; *n* = 7 of 26), and West African countries (15%; *n* = 4 of 26). A high range of years at school was reported by the clients (M = 8.1, SD = 5.0, range 0–18). For a more detailed description of the sample characteristics, see Table 1. Clients reported the experience of M = 7.2 (SD = 2.2, range 3–11) different traumatic event types. At the beginning of treatment, all clients fulfilled the criteria of a PTSD. A comorbid major depression was diagnosed in 58% (*n* = 15 of 26).

**Table 1:** Sociodemography

Characteristics	Sample (*N* = 26)
Female sex, No. (%)	10 (39)
Age, *M* (*SD*, range), years	28.8 (12.2, 14–61)
Education, *M* (*SD*, range), years	8.1 (5.0, 0–18)
Region/Country of origin, No.	
Afghanistan	11 (42)
Western Asia	7 (27)
West Africa	4 (15)
Balkan states	2 (8)
Ethiopia	1 (4)
Sri Lanka	1 (4)
Duration of stay in Germany, *M* (*SD*, range), months	19.0 (13.4, 2–57)
Core family members in Germany, No. (%)	12 (50) ^a^
Accommodation, No. (%)	
Refugee accommodation	19 (73)
Private accommodation	7 (27)
Asylum status, No. (%)	
First instance application	14 (54)
Rejection	7 (27)
Recognition	5 (19)
Traumatic events, *M* (*SD*, range), range 0–12	7.2 (2.2, 3–11)
Natural disaster, No. (%)	7 (27)
Accident, fire, or explosion, No. (%)	22 (85)
Physical assault through a family member or friend	16 (62)
Physical assault through an unknown person	22 (85)
Sexual assault through a family member or friend	5 (19)
Sexual assault through an unknown person	10 (39)
Combat or exposure to war-zone	24 (92)
Captivity	17 (65)
Torture	16 (62)
Life-threatening illness or injury	18 (69)
Other unwanted or uncomfortable sexual experience	8 (31)
Other traumatic event	21 (81)

^a^*n* = 24

### Measures

The instruments – conducted in the form of structured clinical interviews and self-ratings – will be presented thematically as follows. The clinical interviews are consistent with those reported in the concurrent study with untreated refugees [11].

#### Sociodemographic data

Sociodemographic data was asked at T0. Questions assessed, among others, age, sex, education, country of origin, duration of stay in Germany, asylum status, accommodation, and the presence of core family members in Germany.

#### Traumatic events

Two different instruments assessing traumatic events were used at T0 because of the change from DSM-IV to DSM-5: The 12-item event checklist of the PTSD Symptom Scale – Interview Version (PSS-I) [55] and the 17-item Life Events Checklist (LEC-5) [56]. Following the procedure described in Kaltenbach et al. (2018) [11], we used the sum score of the overlapping 12 items.

#### PTSD

PTSD symptoms were assessed both in the structured clinical interviews and in the self-ratings. Consistent with the assessment of traumatic events, at T0 two different instruments were used: the PSS-I [57] was used for DSM-IV and the Posttraumatic Stress Disorder Checklist-5 (PCL-5) [58] for DSM-5. Thirty-five percent (*n* = 9) of the interviews at T0 were conducted with the PSS-I and 65% (*n* = 17) with the PCL-5. At all other time points, the PCL-5 was conducted with all clients. The PSS-I assesses PTSD symptoms with 17 items on a 4-point Likert scale, the PCL-5 includes 20 items measured on a 5-point Likert scale. Both instruments show a good validity and reliability [59–62]. The instruments were administered as structured clinical interviews. The severity of symptoms was rated by combining both frequency and intensity following the suggestion of Foa & Tolin (2000) [59]. In the clinical follow-up assessments, the PCL-5 was used consistently. For PTSD diagnosis, we additionally assessed the remaining criteria according to DSM-5 (Criteria F – G). Because of the good psychometric properties of the PCL-5 as a self-rating instrument and its good ability in detecting clinical change, the PCL was used in the self-rating assessments during and after NET [62, 63]. The time frame for which the symptoms were measured was 1 month in all clinical assessments and 1 week in all self-rating assessments. For the self-rating, the PCL-5 was translated in the according languages of the clients (Albanian, Arabic, Farsi, French, Kurdish, Serbian). To guarantee a valid and accurate translation, one experienced translator generated a written translation and another translator generated a blind back-translation. Differences in the translations were intensively examined and the translations were accordingly adjusted.

For calculations including T0, the item scores of the PSS-I were multiplied with 4/3 to make them comparable to the PCL-5 [63]. To impute the missing three items of the PSS-I, the individual mean of the questionnaire was used. We cannot rule out that the use of two slightly differing PTSD instruments and traumatic event checklists in the baseline assessment could potentially have led to a measuring inaccuracy. For calculations, the PTSD sum score (range 0–80) will be used. Cronbach’s α for the clinical ratings is .85 at T0, .83 at F3, and .88 at F6. For the self-rating, it is .93 at T1, .91 at F3, and .94 at F6. The clinical and self-rating version of the PCL-5 correlated at T0 / T1 (r_s_ = .55, *p* = .003) and at F6 (r_s_ = .62, *p* = .008). The correlation at F3 was not significant (r_s_ = .40, *p* = .090). Most of the time, clinical ratings presented slightly lower PTSD symptoms than those found in the self-ratings.

#### Depression

Depression was assessed in the clinical interviews with the Patient Health Questionnaire – 9 (PHQ-9) [64], a widely used instrument with good psychometric properties [64, 65]. It assesses symptom severity with 9 depression symptoms on a 4-point Likert scale. Additionally, the diagnosis of Major Depression according to DSM-5 can be derived from the PHQ-9. The sum score was used for calculations. Cronbach’s α at T0, F3, and F6 is .82, .77, and .87, respectively.

#### Daily functioning

Daily functioning related to the symptoms of PTSD was assessed in the self-ratings before each NET session as well as in the follow-up interviews. It was administered following the PCL-5. Daily functioning was assessed using eight self-constructed items that showed good results in an earlier study [66]. The items assessed impairment related to relationships with family members, relationships with friends, household chores and duties, fun and leisure activities, work, education, general satisfaction with life, and overall functioning in all areas of life. The items were assessed on the same scale as the PCL-5 (5-point Likert scale). The mean of the applicable items for each person was used for calculations. Cronbach’s α is .87 at T1, .62 at F3, and .90 at F6.

#### Life events during and after treatment

At the beginning of each treatment session as well as in the follow-up assessments, clients were asked if they had experienced positively arousing and / or stressful life events since their last session. If they responded yes, clients were additionally asked to name the event(s) that happened. Retrospectively, categories were generated by clustering the clients’ answers. Findings on life events during and after the treatment are reported separately because of the different time frames.

#### Content of the treatment

The therapist documented the content of each session. The following categories were specified: Narration on negative event, narration on positive event, “lifeline”, psychoeducation, counseling, others. Additionally, therapists wrote down the narration of the client (including the type of event exposed in the session, as well as detailed information about it).

### Data analysis

The programs SPSS 24.0 and R 3.3.2 were used for the data analysis. Variables used for calculations met the preconditions for parametric analysis [67]. Accordingly, t-tests and repeated-measures analyses of variance (ANOVA) were calculated. In case the assumption of sphericity (tested with Mauchly’s test) was not met, the Greenhouse-Geisser correction was used. Generalized eta squared (η_g_^2^) and Hedges’s g were used as effect sizes. To prevent alpha-inflation in case of multiple comparisons, the Bonferroni-Holm correction was applied for post-hoc analyses of ANOVAs. Due to the small sample size, all calculations for influencing factors and the subgroups are exploratory. For comparisons of the symptom trajectories during NET, we summarized the self-rated PTSD symptoms for calculations in five comparable segments. As reference value, we used the first self-rating measurement T1, with the PTSD symptoms being reported directly before the first NET session. Followingly, we parted the other sessions into quartiles (Q25, Q50, Q75, Q100) and calculated the means of the PTSD symptoms in the respective quartiles.

Two change indexes depicting symptom changes in the individuals were used for calculations: (a) The reliable change index (RCI) [68] was used to calculate meaningful symptom changes between the beginning and the end of the treatment, and the follow-up assessments. For the clinical interviews, RCIs between T0 and F3 and F6 were calculated. For the self-rating, the RCIs between T1 and the end of the treatment as well as F3 and F6 were calculated. To calculate the RCIs, the test-retest reliabilities of *r* = .82 for the PCL-5 [69] and *r* = .84 for the PHQ-9 [70] were used. For the standard deviation, the according SD of the baseline assessment was used. If the difference exceeded the threshold with α = .05, the improvement (Z ≥ 1.96) or worsening (Z ≤ − 1.96) was considered as statistically significant. (b) To detect symptom changes during treatment, a less strict criterion - a threshold of 10 points of change suggested by [71] - was used. The 10-point-change was used to organize clients into response groups. Therefore, change scores were calculated by subtracting the first point in time from the subsequent point in time. Accordingly, negative numbers mean a decrease in symptoms, while positive numbers mean an increase in symptoms. To summarize distinct symptom courses, fast responders were classified as those with at least 10 points of change between T1 and Q25 and slow responders with at least 10 points of change between T1 and T75. Clients who did not show more than 10 points of change over the course of the treatment were classified as those not showing an immediate response.

The effect of the exposure of the most distressing event on the self-reported PTSD symptoms was analyzed by extracting the time point right before the exposure of the most distressing event, the time point after the first exposure of the most distressing event, the time point after the last exposure, and the subsequent session. In case a client named more than one most distressing event, the first one was chosen.

To examine the effect of negative life events on the self-reported PTSD symptoms, we extracted the time point a negative event was reported as well as the most proximate time point ahead of the negative experience. Several negative events of one person were used as long as there was at least one time point without a negative event in between. When several negative events happened in a row, we used the first one for the analysis.

Missing values were replaced through multiple imputation (MI) with the R package mice 2.30 [72]. MI is commonly used to estimate missings in multilevel data [73]. It requires the missing data to be missing completely at random (completely unrelated to the data) or missing at random (not related to the missings but to other variables) [74]. Through visual inspection, we can conclude that the data are missing at random. The only systematic missingness was found at T0 in the PSS-I (3 missing items) – therefore, these items were imputed with the individual mean. At item level, missings were replaced if less than 10% of the questionnaire was missing, a higher missingness led to an exclusion of the according questionnaire. In case of missing time points, only the self-rated questionnaires assessed during NET were replaced through MI (< less than 10% missingness).

## Results

### Duration and content of treatment

The duration of active treatment was on average M = 161.0 days (SD = 81.9, range 35–372), with the number of sessions ranging between 6 and 29 sessions (M = 14.3, SD = 5.1). The length of treatment and the number of sessions correlated with the amount of different traumatic event types (r_s_ = .49, *p* = .011; r_s_ = .47, *p* = .016). Furthermore, higher self-reported PTSD symptoms were linked to a longer treatment duration (r_s_ = .47, *p* = .016).

The majority of sessions (M = 93%; SD = 8.5%, range 69–100%) contained content of NET (exposure, lifeline, psychoeducation). The exposure of the most distressing events took on average M = 23% (SD = 13.6, range 7–60%) of the total sessions. Other content such as crisis interventions and counseling on current difficulties was included in M = 25% (SD = 18.6, 0–67%) of the sessions. The experience of new traumatic events during treatment correlated with a higher number of sessions (r_s_ = .43, *p* = .029).

### Replication of the effectiveness of NET

Table 2 depicts the mental health scores at the different measurement points. To capture changes in the mental health symptoms before and after NET, we performed repeated-measures ANOVAs. Clinically-assessed PTSD symptoms showed a significant decrease between T0, F3, and F6 (F (2, 30) = 26.55, *p* < .001, η_g_^2^ = .64). Post-hoc tests revealed a significant decrease between T0 and F3 (t (18) = 7.86, *p* < .001, g = 2.00) as well as between T0 and F6 (t (17) = 4.28, *p* = .001, g = 1.47).

**Table 2:** Mental health scores at baseline, as well as at and 3 months and 6 months follow-up

	T0/ T1	End of NET	F3	F6
Clinical rating				
PTSD sum, *M* (*SD*, range)	43.3 (12.5, 23–66)		20.6 (9.0, 9–48) ^b^	24.9 (12.0, 9–46) ^c^
Depression sum, *M* (*SD*, range)	16.0 (5.8, 5–26)		8.2 (4.6, 1–22) ^a^	10.7 (5.8, 3–23) ^c^
PTSD diagnosis, No. (%)	26 (100)		2 (11) ^b^	7 (39) ^c^
Depression diagnosis, No. (%)	15 (58)		3 (15) ^a^	6 (33) ^c^
Self-rating				
PTSD sum, *M* (*SD*, range)	52.2 (15.5, 22–80)	34.0 (19.7, 2–74)	34.2 (15.0, 8–62) ^a^	38.5 (18.2, 3–62) ^d^
Functionality sum, *M* (*SD*, range)	2.16 (0.9, 1–3.8)	1.37 (0.8, 0–3.3)	1.33 (0.8, .13–3.1) ^b^	1.47 (1.0, 0–3.4) ^d^

*n* = 26, ^a^*n* = 20, ^b^*n* = 19, ^c^*n* = 18, ^d^*n* = 17, PTSD = posttraumatic stress disorder, T0 = clinical baseline assessment, T1 = self-rating in the beginning of the first therapy session, F3 = 3-month follow-up assessment, F6 = 6-month follow-up assessment

Consistently, self-rated PTSD symptoms showed a significant decrease between T1, the last NET session, F3, and F6 (F (3,45) = 12.18, *p* < .001, η_g_^2^ = .45). In detail, the post-hoc tests showed significant symptom reductions between T1 and the last NET session (t (25) = 5.79, *p* < .001, g = 1.01), T1 and F3 (t (19) = 5.71, *p* < .001, g = 1.16), and T1 and F6 (t (16) = 2.76, *p* = .014, g = .81). An increase in PTSD symptoms was found between the last NET session and F6 (t (16) = − 2.59, *p* = .020, g = −.23).

Depression symptoms assessed in the clinical interviews also revealed a significant decline between T0, F3, and F6 (F (2,32) = 12.23, *p* < .001, η_g_^2^ = .43). More precisely, a significant decrease between T0 and F3 (t (19) = 4.74, *p* < .001, g = 1.46) and T0 and F6 (t (17) = 2.95, *p* = .009, g = .90) could be found. Between F3 and F6, an increase in depression symptoms could be detected (t (16) = − 2.41, *p* = .028, g = −.48).

A meaningful symptom decrease in the clinically-assessed PTSD symptoms was found in 63% (*n* = 12 of 19) comparing T0 with F3 and in 44% (*n* = 8 of 18) comparing T0 and F6. Slightly lower numbers were found in the self-rated PTSD symptoms: Compared to T1, 50% (*n* = 13 of 26) showed a meaningful symptom decrease by the end of the treatment, 35% (*n* = 7 of 20) at F3, and 18% (*n* = 3 of 17) at F6. A meaningful decrease in clinically-assessed depression symptoms was found in 60% (n = 12 of 20) and 33% (*n* = 6 of 18) comparing T0 with F3 and F6 accordingly. None of the clients showed a meaningful worsening of PTSD or depression symptoms between the beginning and the end of the treatment and the follow-up time points.

### PTSD trajectories during NET

**Fig. 2:**
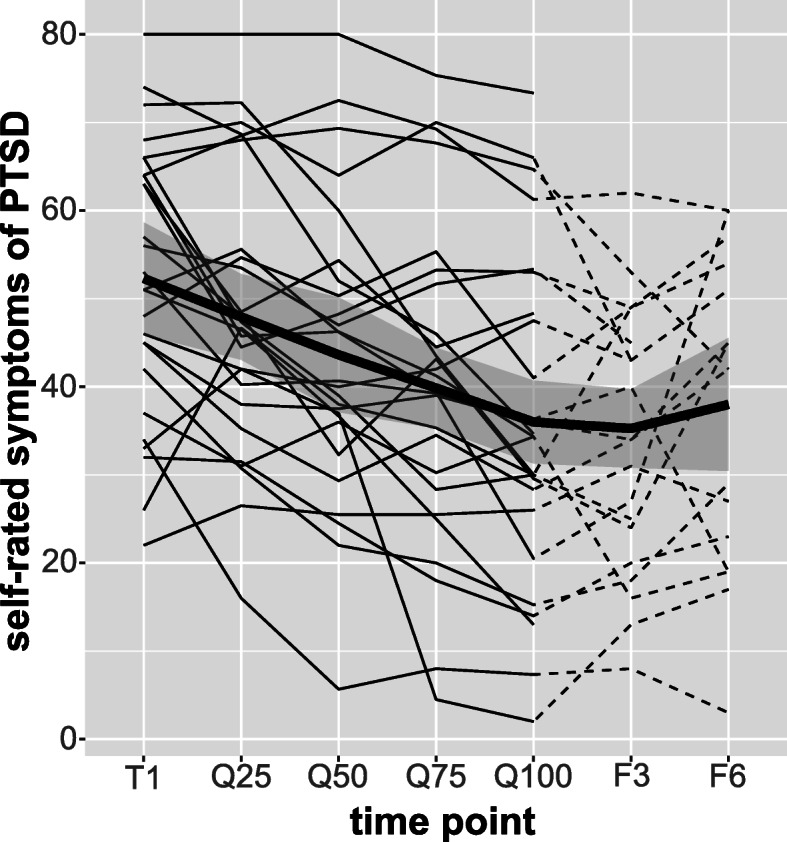
Trajectories of self-rated PTSD symptoms during and after NET. The bold line represents the smoothed overall mean and the grey shade marks the 95% confidence interval. The individual courses are depicted by the thin lines. The dotted lines mark the time after NET. T1 is the beginning of NET, Q25, Q50, Q75, Q100 are the summarized quartiles during NET. F3 and F6 are the 3- and 6-month follow-up assessments

Examining the course of self-rated PTSD symptoms during NET, a significant reduction in symptoms between the first treatment session, the first, second, third, and last quartile of the treatment was found (F (2.14, 53.46) = 18.17, *p* < .001, η_g_^2^ = .42, *n* = 26). Post-hoc tests revealed significant t-tests between nearly all time points (all p < .001, range g = .18–.95). The difference between Q75 and Q100 failed to achieve statistical significance (t (25) = 1.90, *p* = .069). When the follow-up time points were included in the ANOVA, the same effect was found (F (3.60, 54.04) = 7.11, *p* < .001, η_g_^2^ = .32, *n* = 16). Figure 2 shows a graphical depiction of the smoothed average course during and after NET as well as an overview of the individual courses split in quartiles. Individual courses are depicted in Supplementary file 1. Self-rated daily functioning showed a similar pattern, a significant decrease in functional impairment over time was found – both during NET (F (2.24, 56.05) = 15.82, *p* < .001, η_g_^2^ = .39, *n* = 26) and also when including F3 and F6 (F (2.05, 28.65) = 4.73, *p* = .016, η_g_^2^ = .25, *n* = 16).

#### Distinct PTSD symptom courses during treatment

**Fig. 3:**
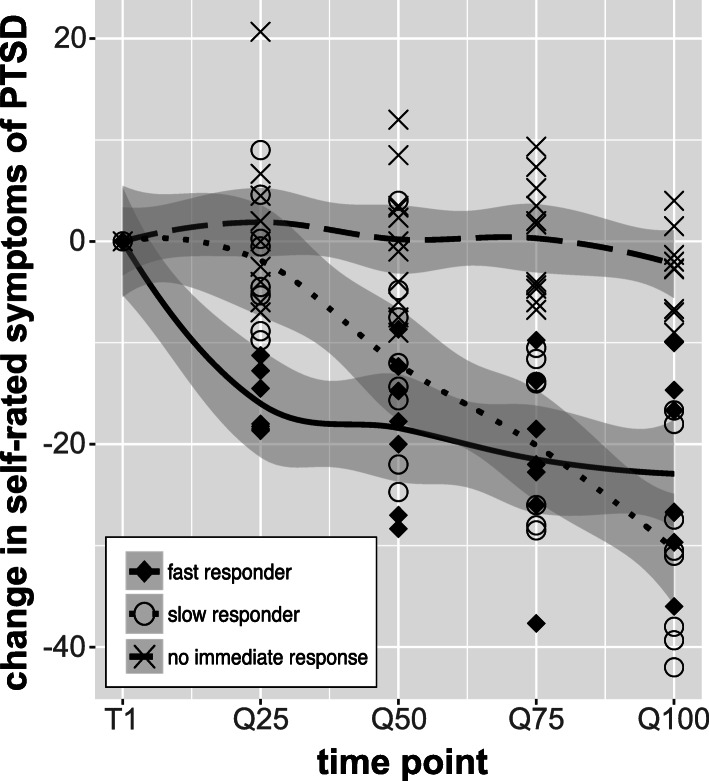
Groups of symptom courses during NET. Fast response is classified as those with a decrease of at least 10 points between T1 and Q25. Slow response is classified as those with a decrease of at least 10 points between T1 and Q75. No immediate response is classified as those who showed a change of less than 10 points over the course of NET. Groups are depicted with the lines, the individual persons are depicted with the according signs. T1 is the beginning of NET, Q25, Q50, Q75, Q100 are the summarized quartiles during NET. Negative values represent symptom improvements in respect to baseline, whereas positive values show aggravations

Clients showed different courses of PTSD symptoms during the course of the treatment. Splitting them in groups with regard to their symptom changes of at least 10 points on the PCL leads to the groups as depicted in Fig. 3. Fast responders showed at least 10 points of change within the first quartile of the treatment (27%, *n* = 7), slow responders showed a change of at least 10 points of change within the first 3 quartiles of the treatment (31%, *n* = 8). Another group resumes those showing no overall change in symptoms at the end of the treatment (42%, *n* = 11). A fourth group would include those with a negative response, meaning a worsening of symptoms of at least 10 points of change over time. Such change was not found in any of the persons. Only one person showed a temporary increase in the symptoms. Sociodemographic characteristics, the number of traumatic events and mental health measures did not differ between responders and non-responders during treatment.

#### The relationship between responding during treatment and responding during the follow-up period

Most of the responders (self-rating) during treatment also showed a relevant PTSD symptom decrease during at least at one of the two follow-up time points (based on the clinically-assessed symptom ratings; 58%, *n* = 7 out of 12). Of the non-responders during treatment (self-report), 78% (*n* = 7 out of 9) showed a delayed response in decreased PTSD symptoms in the follow-up period (based on the clinically-assessed symptom ratings). Only *n* = 2 (out of 21, 9.5%) showed no response in symptoms at any time point.

### Influence of sociodemographic variables and initial symptom levels

No influence of sociodemographic variables such as gender, age, education, family members in Germany, and duration of stay in Germany on the course of PTSD symptoms during and after NET (measured with T1, Q25 – Q100) was found. Further, no influence of the number of different traumatic event types on the change scores could be detected. The initial level of clinically-assessed PTSD and depression symptoms did not correlate with the changes reported during and after NET.

### Influence of imaginal exposure sessions with the most distressing events

The effect of imaginal exposure to the most distressing events on the self-rated PTSD symptoms was examined. No significant difference in symptoms of PTSD before the first exposure of the most distressing event (M = 41.9, SD = 15.9), the following session after the first exposure of the most distressing event (M = 43.5, SD = 17.7), the session after the last exposure (M = 42.4, SD = 17.6), and in the subsequent session (M = 37.9, SD = 19.2) could be found (F(3,75) = 1.70, *p* = .175, η_g_^2^ = .06). On an individual level, 15% (*n* = 4) showed an increase in PTSD symptoms between the first exposure of the most distressing event and the following session, 69% (*n* = 18) showed no symptom change, and 15% (n = 4) showed a symptom decline.

### Influence of current life events during and after treatment

During the period of NET, all but one client (*n* = 25 of 26) reported experiencing at least one negative life event. In total, new negative life events were reported in 24% (*n* = 87 of 371) of the NET sessions. Thereof, 19 events included potentially traumatic content (5%; *n* = 19 of 371). Positive life events were reported in 12% (*n* = 43 of 371) of the sessions. In 27% (*n* = 10 of 37) of the follow-up assessments, clients reported experiencing negative life events; thereof, 6 events were potentially traumatic (16%; *n* = 6 of 37). Positive events were reported in 24% (*n* = 9 of 37) of the follow-up assessments.

Negative life events often named during NET were bad news from relatives or friends living in the home country (*n* = 24 of 87), injury or illness of family members or close friends (*n* = 15 of 87), bad news in the asylum procedure (*n* = 12 of 87), own injury or illness (*n* = 11 of 87), violent verbal or physical argument (*n* = 11 of 87), and violent or illness-related death of a relative or close friend (*n* = 8 of 87). Positive events most commonly named during NET were related to work or school (*n* = 17 of 43), the asylum procedure (*n* = 8 of 43), or celebrations (*n* = 6 of 43). Reports of positive and negative life events at F3 and F6 are comparable to those during NET.

Statistically, PTSD symptoms reported at the time point of a new negative life event during treatment (M = 47.6, SD = 18.7) were significantly higher compared to those at the time point before such a negative event happened (M = 44.2, SD = 19.2; t(63) = − 2.61, *p* = .011, g = .18). In total 64 sessions were counted with a report of a new negative event (including multiple counting within cases) that was not preceded by a previous negative event. In 23% (*n* = 15) of these sessions there was a symptom increase, in 70% (*n* = 45) there was no symptom change and in 6% (*n* = 4) a symptom decrease occurred compared to the previous session. No influence of positive events on the PTSD symptoms could be detected.

## Discussion

The current study tackles the lack of research on symptom trajectories of refugees during and following NET. More than half of the clients showed a symptom decrease during NET and at the 3- and 6-months follow-up assessments. Upon closer examination, individuals showed their major symptom decreases at different time points during NET: fast responders, slow responders, and no immediate response during treatment. However, the distinct symptom courses during treatment were not associated with differential improvements of PTSD symptoms at follow-ups. During treatment, the exposure of the most distressing events did not evoke increases in PTSD symptoms. On the contrary, current stressful life events seemed to aggravate PTSD symptoms.

To our knowledge, this is the first study to systematically examine symptom trajectories of refugees during NET. However, despite the differences within previous reports (with focus on different target populations and TFP approaches), the results of the presented study are in line with the existing research, for example, concerning symptom patterns and their ratio [39, 40]. The relevance of the differing symptom courses during treatment is still unexplained, as there does not seem to be a direct influence of the distinct symptom courses on the long-term mental health outcome after treatment. A possible explanation may lie in the etiological model of the fear network: the recall of traumatic events in its elementary representations (sensations, cognitions, emotions, and interoceptions) embedded in the autobiographical context is thought to segment the fear network systematically. Accordingly, fear networks that have a different composition in different subjects become segmented as memories of particular events with differing speeds during treatment. This leads to distinct symptom courses, albeit, having the same result on the fear network on the long-term. Another reason may lay in genetic and epigenetic factors; especially factors involved in stress regulation seem to influence responsiveness to psychotherapy [75].

Furthermore, the study adds to the limited research base of symptom exacerbations during TFP, confirming that increases in symptoms are only temporarily present in a small percentage of clients and that imaginal exposure does not seem to lead to systematic symptom increases [42, 43] – also and even though TFP was conducted with refugees living in volatile conditions. Concerning this result, the time frame of the symptom assessment (approx. Every 7 days) needs to be considered: symptom increases in the first few days following exposure might not be well detected within the 1 week time frame. However, there are no validated instruments to assess mental health symptoms on a shorter time frame, (i.e.1–2 days after the session). It may not be meaningful to assess PTSD symptoms just during the last 24 h. Rather it might be more preferable to measure physiological components including sleep patterns and perceived stress the day after an exposure session (for example, similar to [44]).

In our study, we found one factor evoking temporary symptom increases – the experience of new stressful life events appears to be connected with higher self-reported PTSD symptoms, even during treatment. This aspect has not been considered in other studies examining symptom trajectories during treatment. However, two multidisciplinary treatment studies with refugees as well as studies examining the natural course of mental health symptoms in refugees also found this negative influence of stressful life events on mental health symptoms [11, 50, 51, 76]. Accordingly, the negative influence of stressful life events on refugees’ mental health seems to be present independent of the type of treatment or treatment at all.

While the majority of clients showed symptom improvements during treatment, 42% of clients did not show significant improvements at the end of treatment. Of those, 78% showed a delayed response during the follow-up period. In line with our outcomes, results of other trauma-focused treatments show that not all clients show symptom improvement after treatment. One reason for delayed symptom improvement may be that the treatment teaches clients a skill to cope with cues triggering the fear network and only when this skill is sufficiently practiced it begins to affect the symptom level. This finding underlies the importance to conduct follow-ups in research but also in clinical settings in order to confirm the benefits from treatment and also to detect delayed responses to treatment. The finding of the slight symptom increase at F6 stays in contrast to earlier studies detecting increasing symptom reductions even 1 year after treatment [23, 27, 77]. This, however, may not be surprising, given the various ongoing postmigrational stressors refugees typically experience in host communities [11, 51], especially social exclusion instead of acknowledgment of human rights violations. In line with this, other mental health difficulties (e.g. depression), potentially triggered through such living conditions, could have had an effect on the PTSD symptoms as well. Nevertheless, none of the clients experienced a meaningful worsening of symptoms from pre-treatment to the end of treatment or the follow-up time points.

The current study has certain implications for clinical practice: Already during treatment, symptom decreases can be detected in around 60% of the clients. Furthermore, the “feared” symptom exacerbations are only present in a small minority of clients and only seem to be of temporary nature. No evidence has been found that these symptom increases are related to the exposure of the traumatic events. It may be that these increases can be partially explained by the stressful life events taking place outside of the therapy setting and it is well possible that these increases had been attenuated due to the treatment. The findings add to the knowledge that TFP in general, but also with refugees living in insecure conditions is well feasible [8, 19, 31, 32]. Postmigrational stressors such as new negative life events led to crisis interventions and counseling in 25% of the sessions in our study, thereby prolonging the duration of treatment. However, the influence of stressful life events does not seem to be dependent on the type of treatment [51]. Accordingly, TFP showing the highest effectiveness among PTSD treatments should be offered to refugees fulfilling PTSD criteria [78]. Concerning the barriers reported by clinicians preventing them from applying TFP [28], training in TFP and thereby disempowering clinicians’ concerns seems to be essential in improving the provision of evidence-based TFP both in inpatient and outpatient settings.

The present study has some limitations that need to be considered: The current study does not offer a comparison group undergoing no or another treatment. However, the concurrent study by Kaltenbach et al. (2018) [11] indicates that there is typically little or no spontaneous remission in samples drawn from the same population. Moreover, the limited sample size does not allow for more complex calculations. Both self-reports as well as clinical interviews are based upon the clients’ subjective reports. Clients handed over their filled self-report questionnaires to their therapists at the beginning of the sessions; this could have had a potential influence on their response pattern. The questionnaire used to assess daily functioning has not yet been validated and showed a low Cronbach’s α at time point F3. Because of the change from DSM-IV to DSM-5 we used two slightly differing PTSD questionnaires for the baseline assessment and had to estimate the missing items for some of the clients. Although the PCL-5 has been validated as a self-rating instrument, validation for refugee populations and the various language versions used in this study is lacking.

## Conclusions

Evidence-based PTSD treatments for refugees – such as NET – are infrequently used in clinical practice due to concerns of potentially negative or destabilizing effects of imaginal exposure. The present study addresses these concerns in depicting not only the long-term benefits of NET, but also examining the PTSD trajectories during treatment. Hereby, the current study as well as previous research with other target groups can lessen these concerns: symptom exacerbations were only found in a small minority, with no evidence that the symptom increases are related to the imaginal exposure. To the contrary, symptom increases seem to be related to stressful life events happening simultaneously to treatment. All of the symptom increases were only temporary, with the majority of refugees showing a fast decline in symptoms during the first sessions or a gradual decrease in symptoms, while others showed no immediate response during treatment. Given the effectiveness of NET as a treatment for PTSD in refugees and the absence of negative side effects during treatment, the use of NET or other TFP approaches in the therapeutical work with refugees can be recommended, even under volatile conditions.

## Supplementary information

**Additional file 1: Supplementary file 1.** Individual trajectories of self-rated PTSD symptoms during and after NET

## Data Availability

The datasets generated during and/or analyzed during the current study are available from the corresponding author on reasonable request.

## Abbreviations

NET: Narrative Exposure Therapy
PTSD: Posttraumatic Stress Disorder
TFP: Trauma-focused psychotherapy
TF-CBT: Trauma-focused cognitive-behavior therapy
CEP: Center for Excellence for Psychotraumatology
T0: Time point of initial clinical assessment
T_1_ - T _xy_: time points of weekly self-reports
F3: 3-months follow-up
F6: 6-months follow-up
DSM-5: Diagnostic and Statistical Manual of Mental Disorders
M: Mean
SD: Standard deviation
PSS-I: PTSD Symptom Scale – Interview Version
LEC-5: Life Events Checklist
PCL-5: Posttraumatic Stress Disorder Checklist-5
PHQ-9: Patient Health Questionnaire – 9
ANOVA: Analyses of variance
RCI: Reliable change index
MI: Multiple imputation
