# Supplementary figures and images for: Trajectories of posttraumatic stress symptoms during and after Narrative Exposure Therapy (NET) in refugees
Source: BMC Psychiatry. 2020 Jun 17;20:312. doi: 10.1186/s12888-020-02720-y (PMC7298826; doi:10.1186/s12888-020-02720-y)

SR PTSD score

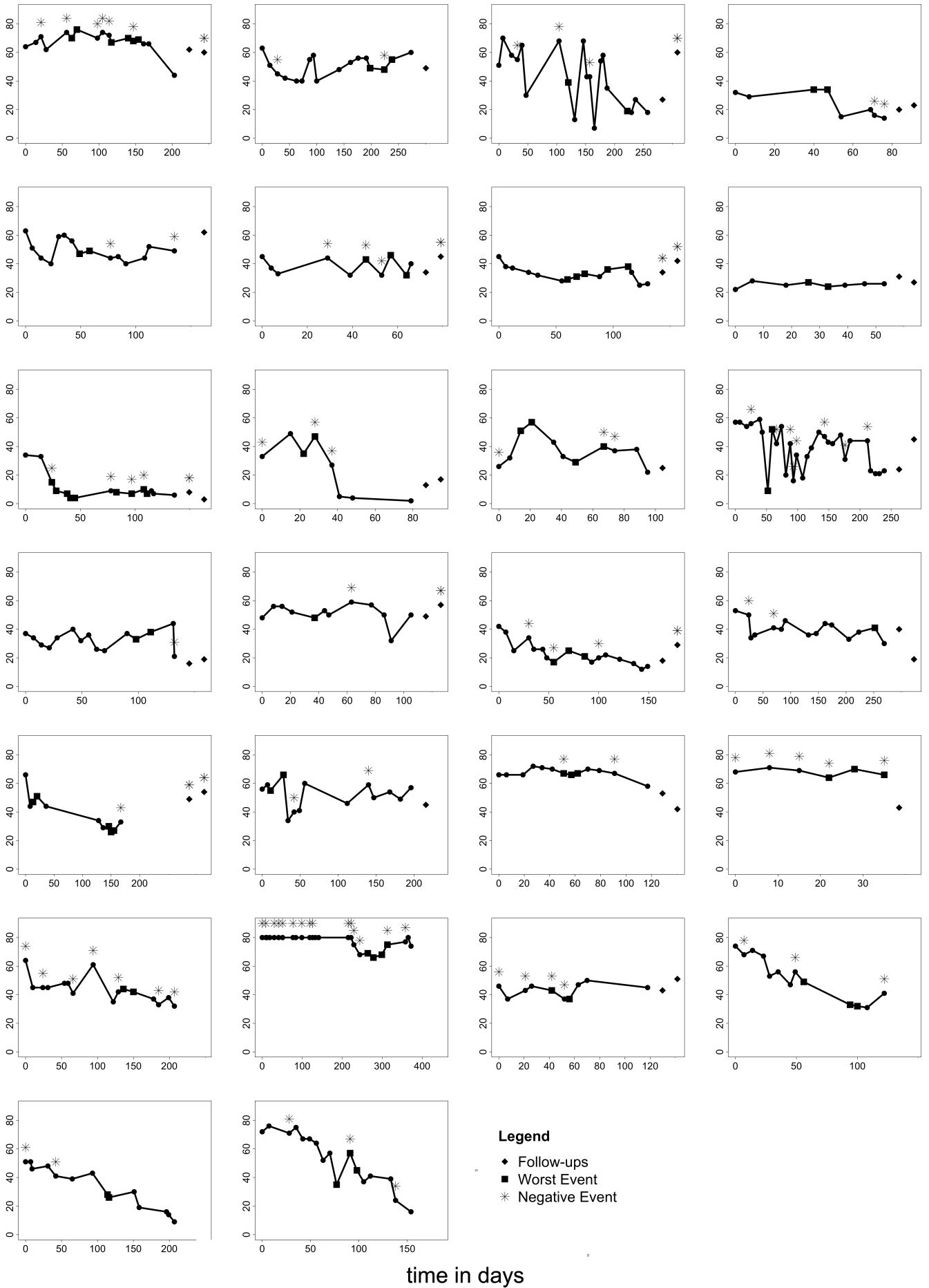

Supplement: Supplementary file 1 — Additional file 1: Supplementary file 1. Individual trajectories of self-rated PTSD symptoms during and after NET [file 12888_2020_2720_MOESM1_ESM.pdf]
